# Risk of Recurrent Coronary Events in Patients With Familial Hypercholesterolemia; A 10-Years Prospective Study

**DOI:** 10.3389/fphar.2020.560958

**Published:** 2021-03-02

**Authors:** Kjell-Erik Arnesen, Ann Vinh Phung, Karoline Randsborg, Irene Mork, Marlene Thorvall, Gisle Langslet, Arne Svilaas, Cecilie Wium, Leiv Ose, Kjetil Retterstøl

**Affiliations:** ^1^ Lipid Clinic, Oslo University Hospital, Oslo, Norway; ^2^ Department of Nutrition, Institute of Basic Medical Sciences, University of Oslo, Oslo, Norway; ^3^ Norwegian National Advisory Unit on Familial Hypercholesterolemia, Oslo University Hospital, Oslo, Norway; ^4^ Institute of Clinical Medicine, University of Oslo, Oslo, Norway

**Keywords:** familial hypercholesterolemia, ASCVD, side effect, TTTFH, statin, PCSK9-inhibitor, ezetrimibe, colesevelam

## Abstract

**Background and Aim:** Real world evidence on long term treatment of patients with familial hypercholesterolemia (FH) is important. We studied the effects of intensive lipid lowering medication (LLM) and optimized lifestyle in the study TTTFH–Treat To Target FH.

**Materials and Methods:** Adults with a first known total cholesterol of mean (95% CI) 9.8 mmol/L (9.5, 10.1) were included consecutively in their routine consultation during 2006. Of the patients 86.4% had a pathogenic FH-mutation and the remaining were clinically diagnosed. We included 357 patients and 279 met for follow-up after median 10.0 (min 8.1, max 12.8) years.

**Results:** Mean (95% CI) low density lipoprotein (LDL-C) was reduced from 3.9 (3.8, 4.1) to 3.0 (2.9, 3.2). More men than women used high intensity statin treatment, 85.2 and 60.8%, respectively. Women (*n* = 129) had higher LDL-C; 3.3 mmol/L (3.0, 3.5), than men; (*n* = 144) 2.8 mmol/L (2.6, 3.0), *p* = 0.004. Add-on PCSK9 inhibitors (*n* = 25) reduced mean LDL-C to 2.0 (1.4, 2.6) mmol/L. At enrollment 57 patients (20.4%) had established atherosclerotic cardiovascular disease (ASCVD), and 46 (80.4%) of them experienced a new event during the study period. Similarly, 222 (79.6%) patients had no detectable ASCVD at enrollment, and 29 of them (13.1%) experienced a first-time event during the study period.

**Conclusion:** A mean LDL-C of 3.0 mmol/L was achievable in FH, treated intensively at a specialized clinic with few users of PCSK9 inhibitors. LDL-C was higher (0.5 mmol/L) in women than in men. In patients with ASCVD at enrollment, most (80.7%) experienced a new ASCVD event in the study period. The FH patients in primary prevention had more moderate CV risk, 13% in ten years.

## Introduction

The introduction of statins in the early 1990s dramatically improved treatment options for patients with heterozygous familial hypercholesterolemia (FH). However, apart from the introduction of ezetimibe, no important new drugs were developed for these patients until the approval of Proprotein convertase subtilisin/kexin type 9 (PCSK9) inhibitors in 2015. Effect and safety of statins is very well documented through large cardiovascular (CV) endpoint studies (Cholesterol Treatment Trialists’ (CTT) et al., 2010). No such CV end point studies exist to document effect in patients with FH, since in these patients, it would be unethical to use placebo. The treatment of FH thus rests on the extrapolation of results of trials in hyperlipidemic patients in the general population (Cholesterol Treatment Trialists’ (CTT) et al., 2010; Ference et al., 2017). Long term prospective cohort studies reporting cardiovascular outcomes are therefore important. In the Simon Broome Register studies (Scientific-Steering-Committe, 1999; Neil et al., 2008; Humphries et al., 2019) analyses were performed before and after statins became available, and excess CHD mortality decreased from 3.4-fold before to a 2.1-fold excess CHD mortality after statins became available (Scientific-Steering-Committe, 1999). Effect of statins in FH was also investigated in a Dutch study reporting that statins decreased risk of CHD by 76% vs. those not treated (Versmissen et al., 2008). Further, several studies on real world data have shown that few FH subjects achieve their LDL cholesterol (LDL-C) treatment goals (Bogsrud et al., 2019; Duell et al., 2019; Iyen et al., 2019; Perez de Isla et al., 2019). In the most recent of these studies mean LDL-C was decreased to 2.90 mmol/L in specialized lipid clinics (Duell et al., 2019). This included, however, treatment with PCSK9 inhibitors in 30% of the patients.

The aim of the present study was to reduce LDL-C to treatment goals as recommended in guidelines in the period 2006–2018, using statins, ezetimibe, resins and diet and lifestyle treatment at study start, and at the end of the study PCSK9 inhibitors in 7.5% of the patients.

## Materials and Methods

**FIGURE 1:**
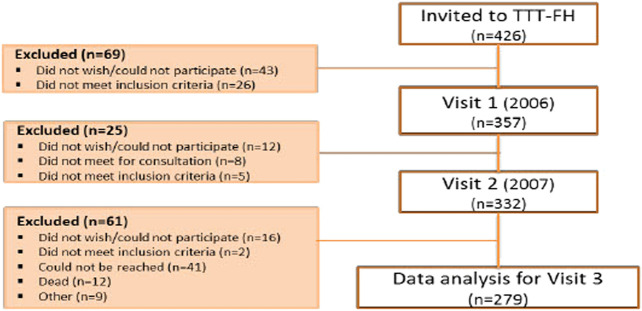
Flow chart.

During January to July 2006, all FH patients between the age of 18–75 (n = 426) meeting for a routine consultation at the Lipid Clinic (LC) were consecutively invited to participate in the study TTTFH–Treat To Target Familial Hypercholesterolemia. Inclusion and exclusion criteria are given in Table 1. Of 426 patients invited, 357 signed an informed consent at visit 1 (V1). One year later, 332 patients met for the second visit (V2). From 2015 to 2019, median 10 (min 8.1, max 12.8) years later 279 patients met for the third and final visit (V3). Data from the 279 patients who completed the full study period are presented in this report. The most common reasons for exclusion were 1) patient could not be reached (n = 41); 2) did not wish to/could not participate (n = 28); 3) death (n = 12). (Figure 1, flow chart). The diagnosis was verified by genetic testing at 86.4% of the patients or clinically by the Dutch Lipid Clinic Network (DLCN) criteria; definite FH at 6%, probable 5% and possible FH at 2.5% (Nordestgaard et al., 2013). Patients underwent a physical examination and a review of diet and lifestyle. Blood samples were analyzed for the routine biological analyses at the Department of Medical Biochemistry, Oslo University Hospital. In few cases blood samples provided by their RGP were used. Medical history was retrieved from the study case report form and the patients’ medical records. Follow-up data was collected after 1 year and at end of study after median 10 years (Figure 1). The diagnosis of metabolic syndrome (MetS) was based on the criteria from NCEP/ATP III (Grundy et al., 2004). In 2006 and 2007 the study was approved by the Regional Ethics Committee as a quality assurance study. The informed consent that was used in 2006 needed to be replaced with a new in 2015, because then the study was approved as a research study. We follow the principles of Good Research for Comparative Effectiveness when possible (Benchimol et al., 2015; Dreyer et al., 2016) using a study protocol with a broad and pre-specified aim.

**TABLE 1:** Inclusion and exclusion criteria.

Inclusion criteria
** **i. Age 18 to 75
** **ii. Signed informed consent
** **iii. Confirmed genetic FH or by Dutch Lipid Clinic Network (DLCN) criteria
Exclusion criteria
** **i. Participating in other on-going study
** **ii. Receiving LDL apheresis
** **iii. Dropping out of scheduled consultations during the half year
** **iv. Not able to fill out questionnaires or join telephone interviews
** **v. Not willing to participate
** **vi. Serious concomitant disease e.g. malignant disease

### Routine Biochemistry Measurement

Total cholesterol (TC), HDL cholesterol (HDL-C), LDL-C, triglycerides (TG), apolipoprotein A1 (ApoA1), apolipoprotein B (apoB), glucose, Glycated hemoglobin (HbA1_c_), C-reaction protein (CRP), aspartate aminotransferase (ASAT), alanine aminotransferase (ALAT), Creatine kinase, Gamma GT and lipoprotein (a) (Lp (a)) was measured. In case of missing Lp (a) values the highest value was retrieved from medical records. Values for non-HDL-C and ApoB/ApoA1 ratio were calculated. Measurements at enrollment and end of study are given in Table 2. The patients’ first measured TC and LDL-C values were retrieved from their medical records.

**TABLE 2:** Measurements at enrollment and at end of study.

		Study start	Study end	P*
	n	Mean (95% CI)	Mean (95% CI)	
Total-C, mmoL/L	272	5.7 (5.5, 5.9)	4.9 (4.7, 5.1)	**<0.001**
HDL-C, mmoL/L	271	1.4 (1.3, 1.4)	1.4 (1.3, 1.4)	0.530
LDL-C, mmoL/L	272	3.9 (3.8, 4.1)	3.0 (2.9, 3.2)	**<0.001**
TG, mmoL/L	268	1 (1, 1.1)	1.2 (1.1, 1.2)	**<0.001**
Non-HDL-C, mmoL/L	270	4.3 (4.1, 4.5)	3.5 (3.3, 3.6)	**<0.001**
ApoA1 g/L	251	1.4 (1.3, 1.4)	1.5 (1.4, 1.5)	**<0.001**
ApoB g/L	252	1 (1, 1.1)	1.1 (1.0, 1.1)	0.122
ApoB/ApoA1 ratio	251	0.78 (0.74, 0.82)	0.74 (0.71, 0.78)	0.101
Glucose, mmoL/L	217	5.1 (5, 5.2)	5.5 (5.3, 5.7)	**<0.001**
HbA1c, %	195	5.4 (5.3, 5.5)	5.7 (5.6, 5.8)	**<0.001**
Systolic BP, mmHG	163	129 (126.8, 131.1)	128.6 (127, 130.4)	0.413
Diastolic BP, mmHG	163	78.2 (76.8, 79.6)	77.2 (76, 78.5)	0.824

*p < 0.05 is considered statistically significant differences between enrolment and end of study. Tested with paired samples T-test. Significant values in bold. Corrected for multiple testing using False Discovery Rate.

### Diet and Body Mass Index

All patients received dietary counseling at each visit, and at least once they consulted a clinical nutritionist. Visit 3 (V3) was an ordinary medical consultation by the MDs in the study, followed by consultations of students in clinical nutrition. We used a validated questionnaire (SmartDiet®) to evaluate the patients’ diet (Svilaas et al., 2002). Body mass index was calculated at enrollment and at end of study.

### Adverse Effects

Adverse effects of the LLM were classified according to the likelihood of whether the effects were caused by the LLM. They were classified as definite, probable, or possible. Adverse effects were classified as definite if it disappeared after termination of the medication and in addition reoccurred at re-challenge with same medication in similar dose, and in addition to that a re-challenge procedure was repeated at least twice. Less certain adverse side effects were classified as probable or possible by clinical assessment.

### Cardiovascular Event and Death

Patients who died (n = 12) were not included in the analysis since we did not have access to the death certificates. Of those who died, eight were male and four females. Median age (95% CI) at time of death was 64.8 (47.3, 67.5) years, the youngest being 38.3, and the oldest 76.1 years. From the hospital medical records, we found that cause of death was myocardial infarction in three patients. One patient died in a traffic accident, one by suicide and in the remaining 7 deaths the cause was unknown. Two of these patients had established atherosclerotic cardiovascular disease (ASCVD) and three patients had cancer.

The number of ASCVD events presented in this study were retrieved from the study case report forms and from medical records. The most common events were percutaneous coronary intervention (PCI), coronary artery bypass graft (CABG) and acute myocardial infarction (AMI).

### Statistics

We used IBM SPSS Statistics version 25.0 (SPSS Inc, Chicago) for statistical analysis. Continuous variables with Normal distribution were presented as mean and 95% confidence interval (95% CI), while skewed variables were presented median and 25^th^–75^th^ percentiles (25–75p) or minimum and maximum values (min-max). Categorical variables were portrayed as number of cases and percentages. Student’s t-test, either independent or paired, were used when comparing normally distributed continuous variables, and Mann-Whitney U test or Wilcoxon signed rank test in case of non-normally distributed variables. Differences between categorical variables were tested using Chi square test for independence, or Fisher’s exact test if the assumptions for using Chi-scare test were violated. A *p*-value <0.05 was considered statistically significant. Analysis of variance (ANOVA) was used to test for statistical significance for three or more groups. Stratification was used as the main method to adjust for factors like sex or types of treatment. Tests were performed both with and without outliers. In case that removal of outliers affected the significance of the results, this was noted in the tables or the text.

## Results

Measurements in the study population at enrolment and end of study are given in Table 2. Measurements at end of study according to use of statins, ezetimibe, resins or additional treatment with PCSK9-inhibitors are given in Table 3. In 86.4% of the patients a FH mutation was identified. The remaining patients were diagnosed according to the DLCN diagnostic criteria for FH (Haase and Goldberg, 2012). Criteria of definite clinical FH (score >8 points) was fulfilled in 6.1%, probable FH (6–8 points) in 5.0% and possible FH (3–5 points) in 2.5%. In 66 patients (24.4%), the diagnosis of FH was made after the age of 40 years (Table 4). Of the 66 patients with late diagnose there were 40 women and 26 men. In the total cohort, mean age of first known high TC measurement was 27.9 years, and only 30.0% of the patients were diagnosed with FH before the age of 20 years.

**TABLE 3:** Measurement at end of study in patients using traditional treatment or PCSK9-inhibitor add-on.

	Total			Traditional treatment (n=255)			PCSK9 add-on (n=25)			P*
	n	Mean	(95% CI)	n	Mean	(95% CI)	n	Mean	(95% CI)	
Total-C, mmol/L	273	4.9	(4.7, 5.1)	252	5.0	(4.8, 5.2)	21	3.9	(3.3, 4.5)	**0.002**
HDL-C, mmol/L	272	1.4	(1.3, 1.4)	252	1.4	(1.3, 1.4)	20	1.5	(1.3, 1.7)	0.262
LDL-C, mmol/L	273	3.0	(2.9, 3.2)	252	3.1	(2.9, 3.3)	21	2.0	(1.4, 2.6)	**<0.001**
TG, mmol/L	271	1.2	(1.1, 1.2)	251	1.2	(1.1, 1.2)	20	1.1	(0.9, 1.3)	0.595
Non-HDL-C, mmol/L	272	3.5	(3.3, 3.6)	251	3.6	(3.4, 3.7)	21	2.2	(1.6, 2.8)	**<0.001**
Lp(a) mg/L	242	562.2	(478.1, 646.3)	218	527.6	(442.5, 612.7)	21	988.8	(595.1, 1382.5)	**0.004**
ApoAl g/L	260	1.5	(1.4, 1.5)	245	1.5	(1.4, 1.5)	15	1.5	(1.4, 1.7)	0.578
ApoB, g/L	262	1.1	(1.0, 1.1)	246	1.1	(1, 1.1)	16	0.8	(0.7, 1)	**0.004**
ApoB/ApoAl ratio	260	0.74	(0.71, 0.78)	245	0.76	(0.72, 0.79)	15	0.54	(0.42, 0.67)	**0.003**
Glucose, mmol/L	235	5.5	(5.3, 5.7)	221	5.5	(5.3, 5.7)	14	5.5	(5.1, 5.8)	0.786
HbAlc, %	238	5.7	(5.6, 5.8)	226	5.7	(5.6, 5.8)	12	5.4	(5.2, 5.6)	0.203
Systolic BP, mmHG	214	129	(127, 130)	197	129	(127, 130)	17	129	(123, 134)	0.952
Diastolic BP, mmHG	214	77	(76, 79)	197	77	(76, 78)	17	81	(76, 85)	0.101

Traditional treatment is statins and/or ezetimibe and/or colesevelam. *P<0.05 is considered statistically significant differences between the group with traditional treatment and those with PCSK9 inhibitor add-on, tested with Independent samples T-test. Significant values in bold. Corrected for multiple testing using False Discovery Rate.

**TABLE 4:** Measurementas at end of study in female male and in those who initiated treatment >age 20 years.

	Untreated before 40 years (N=68)			Male (N=149)			Female (N=30)			p*
Total-C, mmol/L	66	4.9	(4.5, 5.2)	144	4.5	(4.3, 4.8)	129	5.3	(5.1, 5.6)	**<0.001**
HDL-C, mmol/L	66	1.5	(1.4, 1.6)	144	1.3	(1.2, 1.3)	128	1.5	(1.5, 1.6)	**<0.001**
LDL-C, mmol/L	66	2.9	(2.6, 3.2)	144	2.8	(2.6, 3.0)	129	3.3	(3.0, 3.5)	**0.004**
TG, mmol/L	66	1.3	(1.2, 1.4)	144	1.2	(1.1, 1.3)	127	1.2	(1.1, 1.3)	0.742
Non-HDL-C, mmol/L	66	3.3	(3.0, 3.6)	144	3.3	(3.0, 3.5)	128	3.7	(3.5, 4)	**0.006**
Lp (a) mg/L	57	730.7	(472.6, 988.8)	126	591.3	(478.0, 704.7)	113	534.1	(405.1, 663.1)	0.598
ApoA1 g/L	63	1.6	(1.5, 1,6)	133	1.4	(1.3, 1.4)	127	1.6	(1.5, 1.6)	**<0.001**
ApoB g/L	64	1.0	(0.95, 1.1)	135	1.0	(0.97, 1.1)	127	1.1	(1.0, 1.2)	0.071
ApoB/ApoA1 ratio	63	0.70	(0.6, 0.7)	133	0.76	(0.71, 0.8)	127	0.73	(0.68, 0.78)	0.298
Glucose, mmol/L	55	5.8	(5.4, 6.3)	119	5.7	(5.4, 5.9)	116	5.4	(5.1, 5.7)	0.243
HbA1, %	59	5.7	(5.6, 5.8)	123	5.8	(5.6, 6.0)	115	5.6	(5.5, 5.7)	**0.031**
Systolic BP, mmHg	43	131	(126, 136)	116	131	(128, 133)	98	126	(124, 129)	**0.016**
Diastolic BP, mmHg	43	78	(75, 81)	116	79	(78.2, 81.1)	98	75	(73.6, 76.9)	**<0.001**

*p < 0.05 is considered statistically significant differences between male and female, tested with Independent samples T-test. Significant values in bold. Corrected for multiple testing using False Discovery Rate.

### Recurrent and First Time ASCVD Events

At enrollment 57 patients (20.4%) had established ASCVD and 46 (80.4%) of them experienced a recurrent event during the follow-up period of 8–12 years (Table 5). Of those 222 (79.4%) without ASCVD a new event occurred only in 29 (13.1%) during the study period. Among patients diagnosed after 40 years of age, 76.5% had ASCVD at follow-up, as compared to 27.0% among those diagnosed before age of 40 years. As shown in Table 5, patients with recurrent events were older and had significantly higher LDL-C at first time measurement than patients with only one event, and those with one event were older than those without ASCVD. HDL-C and TG did not differ significantly between the groups. Patients with recurrent ASCVD had higher levels of Lp (a) and HbA1c, higher age, and higher prevalence of diabetes, hypertension, smoking and FH diagnosed after 40 years of age (Table 5). The average age at first ASCVD event in the total cohort, was 47.6 years which is in the same range as observed in several other studies (Mata et al., 2011; degoma et al., 2016; Mundal et al., 2016).

**TABLE 5:** Comparison between patients with only one ASCVD event, multiple ASCVD events and those free of ASCVD at end of study.

	NO ASCVD			ASCVD once			Recurrent ASCVD		
	N			N			N		
MI	193	27	(26.3, 27.7)	40	27.3	(25.5, 29.1)	46	27.4	(25.9, 28.9)
Current smokers	186	25	(13.4%)	38	4	(10.5%)	45	9	(20.0%)
Former smokers	166	65	(39.2%)	35	20	(57.1%)	44	24	(54.5%)
	n	mean	(95% CI)	n	mean	(95% CI)	n	mean	(95% CI)
Age at last follow-up^abc^	193	51.3	(49.5, 53.1)	40	60.5	(57.1, 63.9)	40	66.7	(64.3, 69.2)
Age first known total-C^ac^	192	25.2	(23.2, 27.2)	39	32.2	(27.5, 36.9)	39	35.5	(32.6, 38.4)
Age at first ASCVD^c^				38	51.8	(48.3, 55.3)	45	44.0	(40.9, 47.2)
Blood parameters									
Total-C, mmol/L	190	5	(4.8, 5.2)	39	4.5	(4.1, 5)	44	4.7	(4.3, 5.1)
HDL-C, mmol/L	190	1.4	(1.4, 1.5)	39	1.3	(1.2, 1.5)	44	1.3	(1.2, 1.5)
LDL-C, mmol/L^a^	190	3.2	(3.0, 3.3)	39	2.5	(2.2, 2.9)	44	2.9	(2.5, 3.2)
TG, mmol/L^b^	189	1.1	(1.1, 1.2)	39	1.2	(1.1, 1.4)	43	1.3	(1.2, 1.5)
Lp(a), mg/L^b^	161	454	(382, 526)	37	673	(385, 962)	41	898	(593, 1202)
Glocuse, mmol/L^ab^	163	5.3	(5.1, 5.5)	33	6.1	(5.4, 6.8)	38	6.1	(5.5, 6.7)
HbA1c, %^ab^	166	5.6	(5.5, 5.7)	33	5.8	(5.6, 6.1)	39	6.1	(5.8, 6.5)
									
Co-morbidities	N	n	%	N	N	%	N	n	%
Mets^ab^	193	24	(12.4%)	40	16	(40%)	46	18	(39.1%)
Diabetes^b^	193	14	(7.3%)	40	5	(12.5%)	46	9	(19.6%)
Hypertension^abc^	193	22	(11.4%)	40	21	(52.5%)	46	35	(76.1%)
									
Lipid lowering Medication									
High intensity statin therapy	193	132	(68.4%)	40	33	(82.5%)	46	41	(83.1%)
Moderate intensity statin therapy	193	43	(22.3%)	40	5	(12.5%)	46	2	(4.3%)

^a^ Significant difference between «No ASCVD» and «ASCVD once».

^b^ Significant differences between «No ASCVD» and «Recurrent ASCVD once».

^c^ Significant differences between «ASCVD once» and «Recurrent ASCVD». Tested with ANOVA with Bonferroni correction, or fisher’s exact test (2 sided Sign level p < 0.05).

### Metabolic Syndrome

In those who suffered from METS during the whole observation time 68.4% had CVD, and among those who had developed METS at last visit 53.3% had CVD. In those with no METS over the ten-year period 23.3% suffered from CVD, and those who normalized their METS during the observation period 27.3% had CVD (Table 5).

### Lipids and Lipid Lowering Treatment

Mean TC, LDL-C, and non-HDL-C were significantly reduced from start to end of study as shown in Table 2. BMI increased from mean (95% CI) 26.2 (25.5, 26.9) to 27.3 (26.7, 28) kg/m^2^, but blood pressure remained unchanged during the period (Table 2). At the end of the study period, 73.8% of all patients used high intensity statins and 47.8% used add-on ezetimibe (Table 5). PCSK9 inhibitors were used by 9%, as it became available during the last years of the study period. The 25 patients using PCSK9 inhibitors had even lower mean LDL-C (95% CI) 2.0 (1.4, 2.6) (Table 3). Overall, LDL-C ≤1.8 mmol/L was achieved only in 22 patients, representing 8% of the patients in the cohort.

### Adverse Effects

Adverse effects of the LLM were mostly associated with muscle pain for statins, and GI problems for colesevelam. In total, adverse effects were reported by 123 patients (44.1%). The study physicians classified them as definite in 44 patients (16.5%), and 23 patients (8.3%) could not use statins at all due to adverse effects. Characteristics of those not using statins is given in Table 6. Five of the statin intolerant patients used ezetimibe, two used colesevelam and one used PCSK9-inhibitor. In total, 18 patients (6.5%) did not use any kind of LLM, 13 females and five males. Reason for not using LLM was adverse effects in 10 patients, six patients did not report any specific reasons, four women tried to become pregnant and three patients were skeptic to statins.

**TABLE 6:** Measurements according to use of lipdi lowering medication at end of study.

	Patients not using lipid lowering medication (n = 18)			Patients not using statins (n=23)			patients using lipid lowering medication (n = 256)			P*
		n (%)			n (%)			n (%)		
Women		13	(72 2%)		16	(69.6%)		114	(44.5%)	**0.028**
Secondary prevention		3	(16.7%)		5	(21.7%)		104	(40.6%)	0.260
	n	Mean	(95% CI)	n	Mean	(95% CI)	n	Mean	(95% CI)	
Age at V3, years	18	48.3	(40.8, 55.9)	23	49.0	(42.1, 55.8)	256	55.7	(54.2, 57.3)	**0.017**
BMI, kg/m^2^	18	25.4	(23.5, 27.3)	23	25.9	(23.6, 28.1)	255	27.2	(26.6, 27.9)	0.229
First measured total-C, mmol/L	18	9.4	(8.4, 10.5)	23	9.6	(8.7, 10.6)	249	9.8	(9.5, 10.1)	0.681
First measured LDL-C, mmol/L	12	6.6	(5.3, 7.9)	14	6.9	(5.6, 8.3)	121	7.5	(7.1, 7.9)	0.361
Lipdi at V3										
Total-C, mmol/L*	17	7.6	(6.4, 8.7)	22	7.5	(6.5, 8.5)	251	4.7	(4.5, 4.8)	**<0.001**
HDL-C, mmol/L	17	1.4	(1.2, 1.6)	22	1.5	(1.3, 1.7)	250	1.4	(1.3, 1.4)	0.022
LDL-C, mmol/L*	17	5.6	(4.6, 6.6)	22	5.6	(4.7, 6.4)	251	2.8	(2.7, 2.9)	**<0.001**
TG, mmol/L*	17	1.0	(0.8, 1.2)	22	0.9	(0.8, 1.1)	249	1.2	(1.1, 1.3)	**0.022**
Non-HDL-C, mmol/L*	17	6.2	(5.1, 7.2)	22	6.0	(5.1, 6.9)	250	3.3	(3.1, 3.4)	**<0.001**
Lp (a), mg/L	15	360.1	(141.5, 578.6)	20	376.7	(203.3, 550.1)	219	581.4	(490.2, 675.5)	0.189
ApoA1 g/L	16	1.4	(1.3, 1.6)	20	1.5	(1.3, 1.6)	240	1.4	(1.4, 1.5)	0.706
ApoB, g/L*	16	1.6	(1.4, 1.8)	21	1.6	(1.4, 1.8)	241	1.0	(0.98, 1.0)	**<0.001**
ApoB/ApoA1 ratio*	16	1.2	(1, 1.3)	20	1.12	(0.98, 1.27)	240	0.71	(0.68, 0.74)	**<0.001**
Glucose, mmol/L	14	5.1	(4.7, 5.5)	17	5.2	(5.8, 5.6)	217	5.6	(5.4, 5.7)	0.254
HbA1c, %*	15	5.3	(5.1, 5.4)	18	5.2	(5.1, 5.4)	220	5.7	(5.6, 5.8)	**0.008**
Systolic BP, mmHG	10	121	(115, 127)	13	121	(116, 126)	200	129	(127, 131)	**0.029**
Diastolic BP, mmHG	10	74	(68, 79)	13	76	(71, 82)	200	78	(77, 79)	0.063

*p < 0.05 is considered statistically significant differences between those on statins (n=256) vs those off stains (n=23) tested with independent samples T-test, Mann-Whitney U-test and Chi square test or Fisher’s exact test. Significant values in bold. Corrected for multiple testing False Discovery Rate.

### Diet and Lifestyle

The patients improved their diet from enrolment to the last visit as measured by SmartDiet score (*p* < 0.05 for all). At end of study they used less butter and/or margarine as spread on bread, less meat as cold cuts, less low-fiber bread, more fish for dinner and more vegetables, although mean score of fruits and vegetables corresponds to a maximum intake of 4 units per day, which is lower than the national recommendations. On the other hand, patients reported eating more high fat cheese (*p* = 0.006) than recommended. Over half of the population was physically active for at least 1.5 h per week at the end of study, and the majority had an alcohol intake between 0–7 units per week, which did not change significantly during the study period. The number of persons who smoked was reduced by 32.0% from enrolment to end of study, but the number of cigarettes smoked per smoker was not reduced.

## Discussion

In the present study, more than 80% of the FH patients with established ASCVD at enrollment experienced a new event over a 10-year period, despite being treated to mean (95%CI) LDL-C 3.0 (2.9, 3.2) mmol/L, which is a 55% reduction compared to the untreated LDL-C of 6.6 (5.3, 7.9) mmol/L. As observed in other trials on treatment LDL-C is much too high in FH, Duell et al. reported 2.9 mmol/L using PCSK9 inhibitors in 30% of the patients (Duell et al., 2019), Langslet et al. reported 3.2 mmol/L in 909 FH patients of whom 47% had ASCVD (Langslet et al., 2020) and in another recent trial on FH patients LDL-C was 4.0 mmol/L on conventional treatment (Raal et al., 2020). Taken together, this suggests that a mean LDL-C around 3.0 mmol/L is about as low as it is possible to achieve in real-world practice in patients with FH. In those with additional treatment with PCSK9 inhibitors, a mean LDL-C around 2.0 mmol/L is achievable. A treatment goal of ≤1.8 mmol/L was achieved in no more than 8% of FH patients, which is in line with other reports (Bogsrud et al., 2019; Duell et al., 2019; Perez de Isla et al., 2019).

Age at diagnosis and treatment start is an important risk factor in FH; high age at diagnosis resulting in high lifetime LDL-C load and high risk of ASCVD (Nordestgaard et al., 2013). Looking at new and recurrent ASCVD events, we observed that patients with recurrent events were younger when they had their first event (mean 44.0 years) as compared to those who experienced ASCVD only once, who had a mean age of 51.8 years. Thus, the atherosclerosis in patients with recurrent events seems to be more aggressive than in those with only one event. Further, those with recurrent events were slightly older at diagnosis (35.5 years) compared with those with ASCVD once (32.2 years), and patients free of ASCVD were even younger at diagnosis (mean age 25.2 years). Recent studies have underlined the importance of additional risk factors to predict ASCVD risk in FH (Martín-Campos et al., 2018; Perez-Calahorra et al., 2019). We observed (Martín-Campos et al., 2018; Perez-Calahorra et al., 2019) a striking relationship between METS and the prevalence of CVD, in those who developed METS during the observation period 53.3% had ASCVD compared to 23.3% in those with no METS. This highlights the utmost importance of diet and physical activity among FH-patients.

The risk of recurrent ASCVD in FH has not been much investigated previously. A Dutch study of 345 FH patients found that in patients with a history of ASCVD the event risk of was almost 30% per year under age 40 years and 15% in patients older than 60 years (Mohrschladt et al., 2004). The high risk of recurrent ASCVD in the present study might be explained by high age at diagnosis of FH (Krogh et al., 2015), and the fact that so few reaches LDL-C treatment target as compared to people without FH. In the general population, a Danish study from 2016 reported that only 11% of patients experiencing a first time myocardial infarction used statins prior to the event (Kulenovic et al., 2016). After the first-time myocardial infarction, these patients receive statins leading to a major reduction in LDL-C. In contrast, many FH patients are already on intensive per-oral treatment prior to the first event, and further lowering of LDL-C may be difficult to achieve without PCSK9-inhibitors. Persistent high LDL-C after a CVD event in patients with FH could explain the risk of recurrent events in FH demonstrated in the present study. However, no more than 29 (13.1%) of 222 the patients who were free of ASCVD at study start, had a first time ASCVD event during the follow-up, underlining the effect of early primary prevention. Taken together, these data suggest that early treatment start is of major importance to reduce ASCVD events.

We observed that women had 0.5 mmol/L higher LDL-C than men (Table 5), and the higher LDL may be related to the previous finding that women with FH had their first ASCVD event as young as men (Mundal et al., 2016). Usually men are younger at first ASCVD but not in this FH cohort (Mundal et al., 2016).

Adverse effects were reported in 44.1% of the patients, and 16.5% had definite side effects as classified by the study physicians. This implies that many patients accept to use statins despite side effects. However, 8.6% of the patients had stopped using statins and 6.5% did not use any kind of LLM despite having a mean (95CI%) LDL-C of 5.6 (4.6, 6.6) mmol/L.

Routinely collected health data, obtained for administrative and clinical purposes without specific a priori research goals, are increasingly used for research (Benchimol et al., 2015; Dreyer et al., 2016; Sherman et al., 2016). The present study is a prospective cohort study with a pre-specified research protocol. However, using real-world data is challenging. Importantly, missing data on specific measurements was common in the present study, as shown in the tables.

In conclusion, LDL-C ≤1.8 mmol/L was achieved in no more than 8% of the patients despite the intense mostly per oral lipid treatment, with only 9% using PCSK9 inhibitors. As much as 80.7% of the patients with ASCVD at study start had recurrent ASCVD event over a period of median 10 years. The FH patients in primary prevention had a more moderate CV risk (13% in ten years). With maximally tolerated LLM without use of PCSK9 inhibitors mean (95% CI) LDL-C was reduced to 3.1 (2.9, 3.3), which is much higher than the recommended levels in patients with established ASCVD or with FH, and especially in the combination of both. This illustrates the need for more extensive use of PCSK9-inhibitors among the FH-patients.

## Data Availability

The raw data supporting the conclusions of this article will be made available by the authors, without undue reservation.
